# Future time orientation, life projects, and career self-efficacy of unemployed individuals

**DOI:** 10.3389/fpsyg.2023.1230851

**Published:** 2023-10-12

**Authors:** Ana Daniela Silva, Catarina Luzia de Carvalho, Vinicius Coscioni, Maria do Céu Taveira

**Affiliations:** School of Psychology, University of Minho, Braga, Portugal

**Keywords:** career self-efficacy, future time orientation, life project, unemployed people, mediation analysis

## Abstract

This study examined the relationship between two variables of the psychological future [future time orientation (FTO) and life project (LP)] and their relationship with career self-efficacy in unemployed individuals. Participants were 216 unemployed adults (151 women, 65 men), aged from 18 to 67 years old (*M* = 42.8, *SD* = 10.57), who responded to measures of distance and impact of future time orientation, identification and involvement in life project and career exploration and decision-making self-efficacy. Results of latent mediation analysis and correlational analysis indicated that there is a direct effect between FTO and LP, but also an indirect (i.e., mediating) effect between them through career self-efficacy beliefs. These findings suggest that unemployed individuals with a stronger sense of future time orientation are more likely to identify and engage with their life projects, and that this organization of their life projects is mediated by their levels of career self-efficacy. Overall, the study provides important insights into the psychological factors that can impact the careers behaviors of unemployed individuals, as well as on the characteristics of career psychological interventions with this public.

## 1. Introduction

1. Individuals across various age groups often worry about their work identity and future (Park et al., 2018). This may explain the importance of employment in boosting psychological resources (e.g., social support and well-being) and quality of life (Bonanomi and Rosina, 2022). Job loss is often an undesirable and uncontrolled event (Panari and Tonelli, 2022) that may change people’s perception of their future (Fidelis and Mendonça, 2021). In turn, long-term unemployment may produce a corrosive effect on individuals, in terms of future planning and job searching competence (Mühlhaus et al., 2021). Likewise, people who have lost their job in the past are more prone to feel pessimistic about potential unemployment in the future, leading to insecurity and unhappiness (Knabe and Rätzel, 2011).
2. Even though psychological future has already been considered within career interventions aimed at developing employability (e.g., Ginevra et al., 2017), the existing literature is still scarce regarding the interrelation between distinct future-related variables in unemployed individuals. Previous research on the psychological future of unemployed people focused on the comparisons with other occupational statuses, namely, students and employees (Parola and Donsì, 2019; Felaco and Parola, 2022; Parola and Marcionetti, 2022). Future orientation has been identified as a moderator of the relationship between employment status and health outcomes (Parola and Marcionetti, 2022) and a mediator of the relationship between employment status and well-being (Felaco and Parola, 2022). In turn, a few qualitative studies have focused on the description of unemployed individuals’ envisions for the future (Daskalaki and Simosi, 2018; Sools, 2020; Mühlhaus et al., 2021).
3. This study advances the existing literature by examining the relationship between two variables of the psychological future in unemployed individuals, future time orientation (FTO) and life project (LP). In addition, it further investigates the relationships between such constructs and career self-efficacy, an important individual resource in the context of job search (Taveira et al., 2017). Therefore, the purpose of this study is to examine the relationships between FTO, LP, and career self-efficacy in unemployed individuals.

### 1.1. Unemployment and job market in the 21st century

1. The context of the job market in the 21st century presents various challenges for individuals transitioning into the world of work at different stages of development. For instance, the shift from college to the professional world involves stages like anticipation, adjustment, and achievement (Wendlandt and Rochlen, 2008). In another instance, as a new worker, it is possible for individuals to experience insecurity, inadequate skills, reduced self-confidence, heightened stress, early job turnover, and the establishment of their roles in a workplace (Tynjälä and Heikkinen, 2011). A further example applies to virtual work settings where work and family roles overlap can lead to boundary conflicts (Shumate and Fulk, 2004). In the 21st-century work environment marked by unpredictability, unemployment emerges as a frequent career transition, bearing substantial repercussions for the individuals undergoing this challenging phase (Di Fabio et al., 2018).
2. The experiences of unemployed people are influenced by their fundamental cognitive beliefs, which affect their capacity to adapt to career-related events (Thompson et al., 2017). The experience of this career transition is also influenced by factors such as age, gender, and education level (Sousa-Ribeiro et al., 2018). Age is a significant factor, with older adults (over 50 years old) experiencing a more adverse impact from unemployment (Kenny and Rossiter, 2017), while younger individuals (ages 18–25 and over 25 years old) often seek new experiences (Bonanomi and Rosina, 2022). Gender disparities also exist, as men are frequently more psychologically affected by unemployment (Chung and Hahn, 2021), while women face greater vulnerability due to societal pressures (Knabe et al., 2016). Higher levels of education generally result in better mental health outcomes, but when highly educated individuals experience unemployment, it can challenge their employability (Möwisch et al., 2021). Conversely, individuals with lower skill levels are at a higher risk of unemployment (OECD, 2022). The duration of unemployment also plays a crucial role, as prolonged unemployment can lead to negative self-perceptions and reduced motivation (Thompson et al., 2017).
3. Despite the influence of these factors, it is widely acknowledged that job loss and unemployment are associated with negative psychological outcomes (Thill et al., 2018). Individuals’ psychological resources are therefore fundamental in enabling them to cope with unemployment. For instance, optimism, hope, and emotional intelligence play a role in navigating career transitions (Di Fabio et al., 2018). Effective coping strategies, such as problem-solving and seeking social support, can also help individuals manage the stress and emotional impact of unemployment (Thill et al., 2018). Social support from family, friends, and professional networks has been shown to buffer the negative effects of unemployment on mental health (Wigand et al., 2019). The challenges of unemployment in the 21st century as well as the psychological resources needed to cope with the unemployment experience are complex and multifaceted. They require a comprehensive understanding of the factors that might enable individuals to cope with the experience of unemployment.

### 1.2. Future time orientation and life projects

1. Two distinct theoretical approaches investigate people’s psychological future through different lenses, the athematic approaches, and the thematic approaches (Seginer, 2009). The athematic approaches focus on relatively enduring psychological features related to how one deals with the psychological future (Gjesme, 1983; Zimbardo and Boyd, 1999; Husman and Shell, 2008). Conversely, the thematic approaches assess the psychological future through its content, i.e., the cognitive representations of the future and their motivational and behavioral components (Nurmi, 1991; Seginer, 2009). The thematic approaches are named thematic because they necessarily refer to themes related to the future, whereas the athematic approaches do not. The two approaches are complementary rather than controverse (Gjesme, 1983; Coscioni et al., 2020).
2. Different psychological constructs have been used to describe the nature of the psychological future. One of those constructs is FTO, defined as “the personal disposition to have current psychological functioning impacted by the psychological future” (Coscioni et al., 2023, p. 3). Another one is LP, defined as “an ongoing evolving process to form, enact, and maintain intentional structures and actions that, altogether, encompass a long-term, meaningful, and prospective narrative capable of guiding decisions and behavior in daily life” (Coscioni, 2021, p. 144). FTO is a relatively enduring personal disposition that impacts how one deals with the future, whereas LP are narratives of one’s intended future. Therefore, FTO and LP, respectively, refer to a personal disposition and a state of people’s psychological future. As a trait, FTO is a personal disposition that impacts behavior and decisions toward the future, such as how one constructs and implements one’s LP (i.e., a specific manifestation of FTO). Thus, the first hypothesis of this study is: (H1) unemployed individuals with higher rates of FTO are more likely to have more coherent LP.

### 1.3. Career self-efficacy of unemployed people

**Figure 1:**
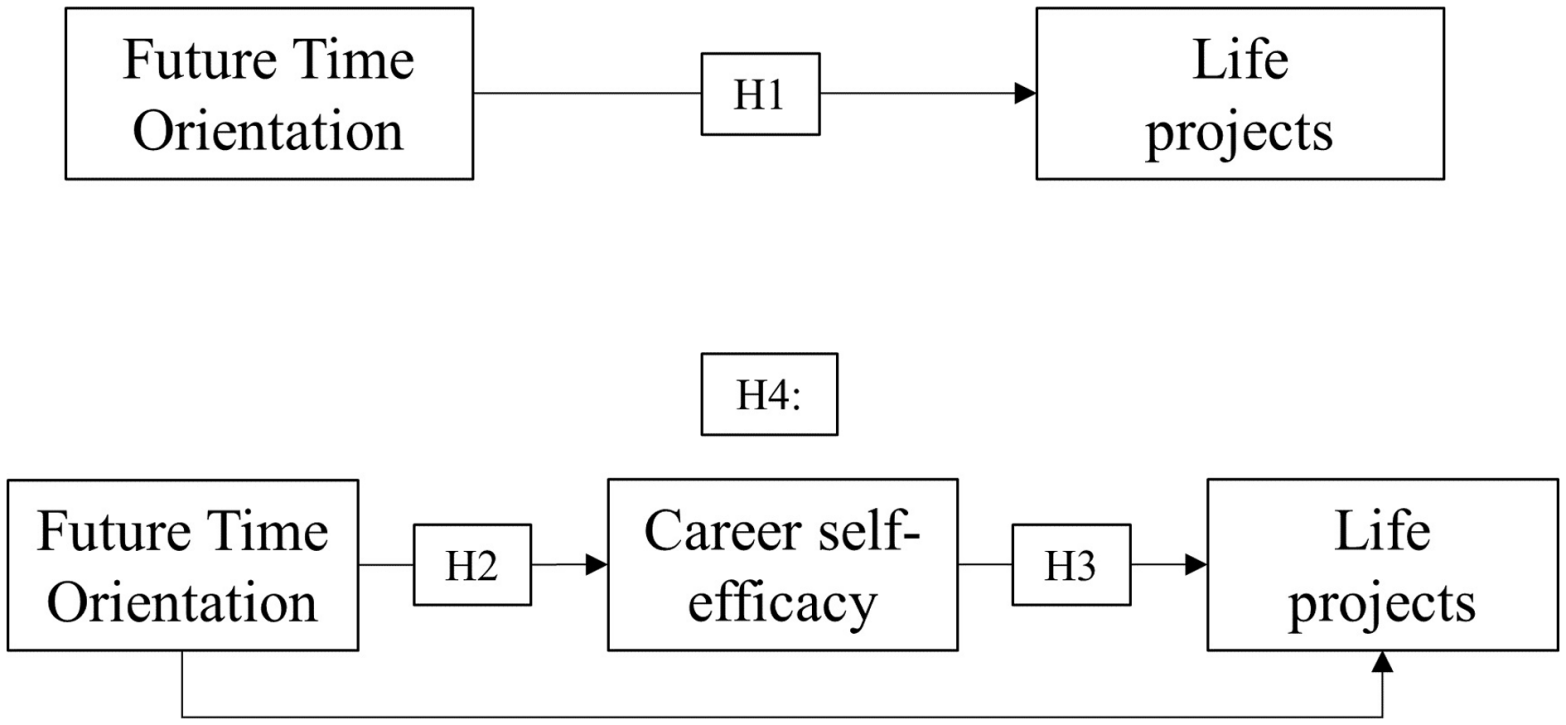
Mediation analysis. n=216. All parameters are standardized and significant at α=0.05. The measurement model is ignored yet all factors loadings were significant and above 0.40.

1. The concept of self-efficacy is fundamental to understanding the careers of people with less access to the information necessary to develop control beliefs, as may be the case with unemployed people (Silva et al., 2009). From a sociocognitive perspective, self-efficacy refers to the individual’s belief in their capability to organize and implement the courses of action required to produce specific attainments (Bandura, 1997). Self-efficacy has been studied within career theories and integrated into the Socio-Cognitive Career Theory (Lent and Brown, 2013). Career development self-efficacy refers to the perception of individuals’ ability to perform specific tasks necessary for career preparation, entry, or adjustment (Lent and Brown, 2013). More recently, Lent et al. (2017) acknowledged two domains of career self-efficacy, namely, career exploration self-efficacy and career decision-making self-efficacy. Career exploration self-efficacy is the perceived ability to identify and engage with information about the self and the environment related to career development, whereas career decision-making self-efficacy is the perceived ability to successfully complete tasks to make significant career decisions (Lent et al., 2016).
2. For unemployed individuals, career self-efficacy is intimately associated with the capacity to change one’s unemployment situation (Creed et al., 2001). For example, job search self-efficacy reflects one’s beliefs about performing tasks that may lead to becoming reemployed, such as creating resumes, looking for potential jobs, interviewing, and networking socially to locate job openings (Maddy et al., 2015). In unemployed people, career exploration self-efficacy is predicted by employability beliefs such as autonomy, striving, optimism, and acceptance of challenges (Taveira et al., 2023). Concerning career decision-making self-efficacy beliefs, unemployed adults have as much confidence in career decision making as other groups (e.g., college students; Bullock-Yowell et al., 2012, 2014).
3. The notion of career also implies a time perspective since future orientation helps individuals define career goals and the pathways to achieve them (Atanásio et al., 2013). Career self-efficacy beliefs are related to beliefs regarding the future (Bandura, 1997); thus, how one deals with the future might impact one’s sense of self-efficacy. Therefore, the second hypothesis of this study is: (H2) unemployed individuals with higher rates of FTO are more likely to have more adaptive career self-efficacy. Considering that self-efficacy also influences how one sets future goals and acts accordingly (Lent et al., 2016), the third and fourth hypotheses of this study are: (H3) unemployed individuals with higher rates of career self-efficacy are more likely to have more coherent LP and (H4) career self-efficacy mediates the relationship between FTO and LP in unemployed people, respectively.
4. The four hypotheses are summarized in Figure 1. The associations between future time orientation and life projects (H1) have already been tested in previous studies (Novak, 2023), yet not specifically with unemployed individuals. Likewise, a study with students has investigated the associations of time perspective and career self-efficacy (H2; Kvasková and Almenara, 2021). To our knowledge, this is the first work testing the associations of career self-efficacy and life projects (H3), as well as the mediation effect of career self-efficacy on the relationships between future time orientation and life projects.

## 2. Methods

### 2.1. Participants and procedures

1. Data collection occurred within the framework of the Careers Project (ALG-06-4234-FSE-000047), a partnership for social impact to support employability in Algarve, Southern Portugal. Algarve is a tourism-dependent region that has witnessed the largest increase in unemployment rates during the COVID-19 pandemic in Portugal (PORDATA: Base de Dados Portugal Contemporâneo, 2021). Over thirty-three thousand residents in Algarve have become unemployed during the pandemic, comprising an increase of over 150% compared to the beginning of the isolation measures (Instituto do Emprego e da Formação Profissional, 2021).
2. Participants were unemployed individuals invited by a governmental institution to attend a career intervention. Those interested in the intervention filled in an online survey, at Qualtrics platform, from April to May 2022. Participants provided their consent form before answering the survey and the research procedures were approved by an ethical commission from Portugal (CEICSH 002/2022).
3. Altogether, 226 people responded to the survey. After data management, 10 participants were removed, of which seven filled in the same response category in several scales, and three had unusual response patterns (i.e., Mahalanobis distance per degree of freedom above 3.0; Hair et al., 1998) in three or more scales out of five. Hence, 216 participants aged from 18 to 67 years old (*M* = 42.8, SD = 10.57) were analyzed. Participants were predominantly female (*n* = 151, 69.9%), nearly one fifth (*n* = 39, 18.1%) were not from Portugal (yet they all were fluent in Portuguese), 42 (19.5%) had not finished high school, 82 (38.1%) had completed high school, and 91 (42.3%) had a college degree.

### 2.2. Measures

#### 2.2.1. Future time orientation scale

The FTOS was created in Portuguese (both European and Brazilian) and English (Coscioni et al., 2023). It is composed of two subscales that measure distance (three items) and impact (five items). Distance refers to perception of distance into the future (e.g., “Two years in the future seems to me like a short period of time”), whereas impact entails the influences of psychological future in current decisions and behavior (e.g., “I value activities that may benefit me in the long run”). Each statement is responded to in a 7-point scale ranging from ‘strongly disagree’ to ‘strongly agree.’ The FTOS properly fit the data,^1^ *χ*^2^ = 21.4, *df* = 19, *p* = 0.315, *RMSEA* [90% C.I.] = 0.024 [0.000; 0.063], *CFI* = 0.990, *TLI* = 0.985, *SRMR* = 0.039. The reliability ranged from acceptable to moderate, *Ω = 0*.66 and *Ω = 0*.77, for distance and impact, respectively.

#### 2.2.2. Life project scale

The LPS was created in Portuguese (both European and Brazilian) and English (Coscioni, 2021). The scale is composed of two subscales that measure identification (four items) and involvement (four items). Identification refers to clearness regarding one’s intended future (e.g., “I am aware of what I want for my future life”), whereas involvement entails the enactment of plans and efforts toward one’s intended future (e.g., “I’m making efforts to achieve what I want for the future”). Items are responded to in a 7-point scale ranging from “totally disagree” to “totally agree.” The LPS properly fit the sample of this study^1^, *χ*^2^ = 44.0, *df* = 19, *p* < 0.001, *RMSEA* [90% C.I.] = 0.078 [0.054; 0.102], *CFI* = 0.960, *TLI* = 0.941, *SRMR* = 0.040. Both factors met good reliability, *Ω = 0*.90 and *Ω = 0*.85, for identification and involvement, respectively.

#### 2.2.3. Career exploration and decision-making self-efficacy scale

The CEDMSES was created in the United States (Lent et al., 2016) and has been adapted to Portugal (Lent et al., 2022). The scale is composed of two subscales that assess career exploration self-efficacy (four items, e.g., “Dealing with disappointment if first choice does not work”) and career decision-making self-efficacy (eight items, e.g., “Identify careers that make the best use of your abilities”). Each item is responded to in a 5-point scale ranging from “totally disagree” to “totally agree.” The scale did not fit the sample of this study^1^, *χ*^2^ = 372.2, *df* = 53, *p* < 0.001, *RMSEA* [90% C.I.] = 0.167 [0.152; 0.183], *CFI* = 0.805, *TLI* = 0.758, *SRMR* = 0.063. Based on the modification indices, four item pairs were correlated, improving the fit indices, *χ*^2^ = 121.1, *df* = 49, *p* < 0.001, *RMSEA* [90% C.I.] = 0.083 [0.066; 0.099], *CFI* = 0.956, *TLI* = 0.941, *SRMR* = 0.052. The reliability met excellent results, *Ω = 0*.92 and *Ω = 0*.83, for career decision-making self-efficacy and career exploration self-efficacy, respectively.

### 2.3. Data analysis

First, the associations between variables were tested considering Spearman correlations of their factors scores computed *via* maximum *a posteriori* method (Bock and Aitkin, 1981). Spearman correlations were selected because Shapiro–Wilk tests suggested the violation of normality. The correlation coefficients were interpreted using the following cutoffs: |*ρ*| < 0.3, weak; 0.3 ≤ |*ρ*| < 0.5, moderate; otherwise, strong (Dancey and Reidy, 2007).

Next, hypotheses were tested by latent mediation analysis (Hayes, 2017) *via* structural equation modeling (Kline, 1998). Maximum likelihood robust (MLR; Satorra and Bentler, 2001) estimator was used because Mardia’s test suggested violation of multivariate normality, *M_skewness_* = 7,524.2, *p* < 0.001, *M_kurtosis_* = 32.5, *p* < 0.001. In addition, according to Rhemtulla et al. (2012), MLR outperforms ordinal estimators in the case of scales with six to seven response categories, such as the FTOS and the LPS. A 2-step approach was implemented (Hayes, 2017). First, a predictor-only model was tested, with FTO regressing on LP. Second, the full mediation model was tested with FTO regressing on LP through career self-efficacy. The indirect effect of self-efficacy was tested considering the bootstrap (500 resampling) 95% confidence intervals. Standardized coefficients were reported to allow for the identification of effect sizes. The following cutoffs were used for interpretation: | *β* | < 0.1, spurious; 0.1 ≤ | *β* | < 0.3, weak; 0.3 ≤ | *β* | < 0.5, moderate; otherwise, strong (Cohen, 1992). As the mediators had a considerable high correlation with each other, alternative models were tested considering each mediator separately.

The models’ goodness of fit was tested by the comparative fit index (CFI), Tucker-Lewis index (TLI), root mean square error approximation (RMSEA), and standardized root mean residual (SRMR). CFI and TLI above 0.950, and RMSEA and SRMR equal or below 0.080 indicate excellent fit (Schreiber et al., 2006). Alternatively, CFI and TLI between 0.900 and 0.950, and RMSEA and SRMR between 0.080 and 0.100 are acceptable (Brown, 2006).

The sample size and number of outliers were appropriate. A sample size calculator (Soper, 2022) suggested the minimum of 89 participants for model structure. In addition, considering *α* = 0.05, *β* = 0.80, and the model structure being tested (i.e., with 28 observed variables and six latent variables) the mediation analysis was powerful to detect significant parameters with an effect of *β* ≥ 0.265. This means the sample was not powerful enough to detect significant effects with a weak effect of *β* < 0.265. As for the outliers, Mahalanobis distance suggested 11 atypical responses. The models were implemented with and without outliers. No big differences were observed with the removal of outliers and, thus, they were retained.

All analyses were implemented in R 4.1.1 (R Core Team, 2020). Correlations were assessed with psych 2.3.3 (Revelle and Revelle, 2015). Structural equation modeling was tested in lavaan 0.6–9 (Rosseel, 2012). The indirect effect of mediators was computed with manymome 0.1.6 (Cheung and Cheung, 2022). Mahalanobis distance was assessed with rstatix 0.7.0 (Kassambara, 2021). Normality was tested with MVN 5.9 (Korkmaz et al., 2014).

## 3. Results

Table 1 displays the descriptive statistics of direct and regression scores, as well as Spearman correlations between FTO, LP, and career self-efficacy. The associations between impact, identification, and involvement were strong. Distance was not correlated to any LP dimensions and was weakly correlated to impact. The two career self-efficacy dimensions were strongly correlated one to the other and moderately correlated to impact, identification, and involvement. Distance was weakly correlated to career exploration self-efficacy and not correlated to career decision-making self-efficacy.

**Table 1:** Descriptive statistics and Spearman correlations.

	Direct scores		Regression scores			Spearman correlations				
	M	SD	SD	Skew.	Kurt.	(2)	(3)	(4)	(5)	(6)
(1) Identification	5.42	1.20	1.13	−0.92	0.44	0.905**	0.152	0.503**	0.481**	0.415**
(2) Involvement	5.40	1.10	0.99	−0.81	0.45		0.128	0.512**	0.405**	0.377**
(3) Distance	4.37	1.17	0.80	−0.61	−0.56			0.293**	0.146	0.252**
(4) Impact	5.90	0.68	0.52	−0.46	0.01				0.394**	0.397**
(5) CD	3.34	0.76	0.67	0.24	−0.35					0.728**
(6) CE	3.05	0.86	0.66	0.24	−0.14					

n = 216, *p < 0.05, ** p < 0.001, CD = career decision-making self-efficacy, CE = career exploration self-efficacy. The mean of regression scores are always zero, as they represent the extent to which participants deviate from the mean.

**Figure 2:**
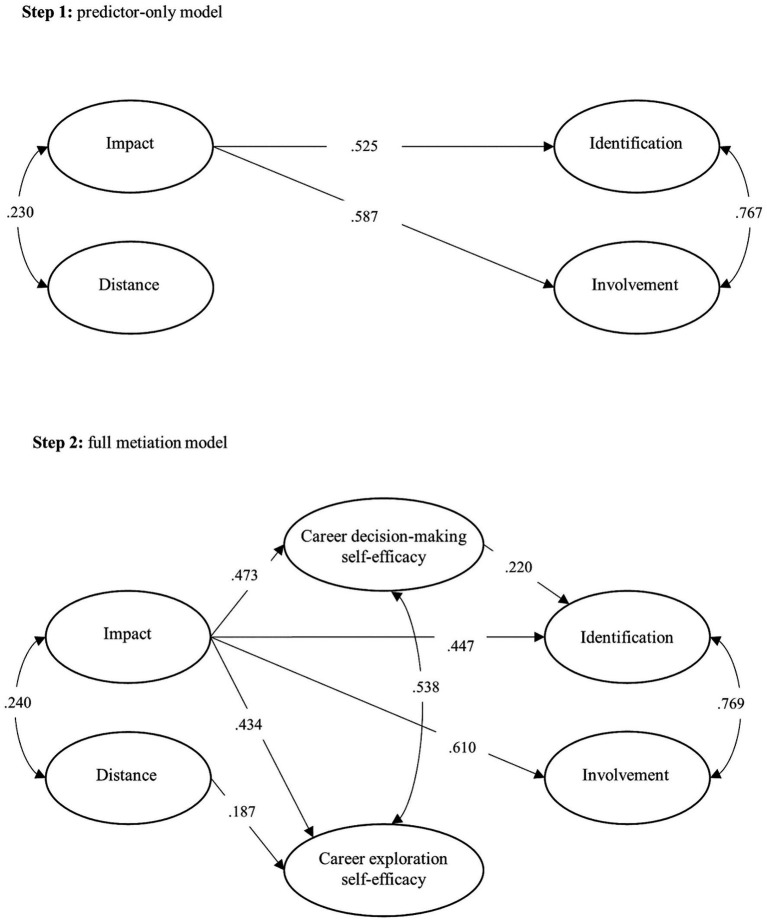
Predictor-only model and full mediation model.

The first step of the mediation analysis (i.e., the predictor-only model) is summarized on the top of Figure 2. The illustrated model is a respecified one after the elimination of non-significant paths. The model fit met excellent RMSEA and SRMR values and acceptable CFI and TLI results, *χ*^2^ = 172.7, *df* = 100, *p* < 0.001, *RMSEA* [90% C.I.] = 0.058 [0.045; 0.071], *CFI* = 0.935, *TLI* = 0.922, *SRMR* = 0.058. Impact was strongly associated with both identification and involvement yet distance was not associated with any LP dimensions. Therefore, the first hypothesis was partially corroborated.

The second step of the mediation analysis (i.e., the full mediation model) is summarized on the bottom of Figure 2. The illustrated model was respecified after the elimination of the non-significant paths, meeting acceptable to excellent fit indices, *χ*^2^ = 533.6, *df* = 338, *p* < 0.001, *RMSEA* [90% C.I.] = 0.052 [0.044; 0.059], *CFI* = 0.939, *TLI* = 0.932, *SRMR* = 0.067. Impact was moderately associated with the two career self-efficacy dimensions yet distance was associated only with career exploration self-efficacy with a weak effect. As for the associations between career self-efficacy and LP, only the path from career decision-making self-efficacy to identification was significant, though with a weak effect. Therefore, the second and third hypotheses were partially corroborated.

**Figure 3:**
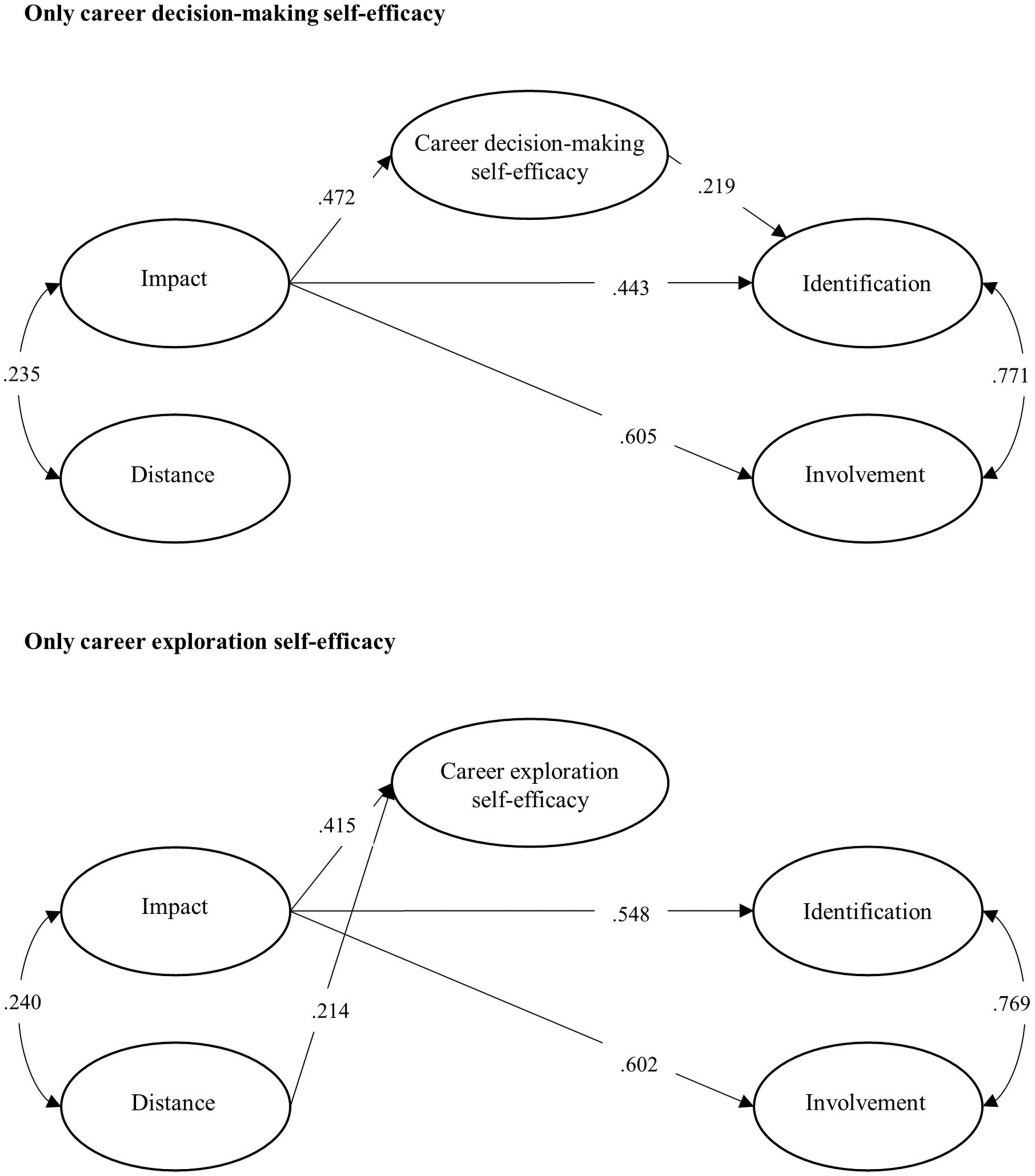
Alternative model.

The alternative models with only one mediator each are displayed in Figure 3. The model with only career decision-making self-efficacy presented excellent fit indices, *χ*^2^ = 402.1, *df* = 244, *p* < 0.001, *RMSEA* [90% C.I.] = 0.055 [0.046; 0.063], *CFI* = 0.936, *TLI* = 0.927, *SRMR* = 0.069. The regression coefficients were very close to those found in the model with multiple mediators. The model with only career exploration self-efficacy also exhibited good fit, *χ*^2^ = 235.2, *df* = 162, *p* < 0.001, *RMSEA* [90% C.I.] = 0.046 [0.034; 0.057], *CFI* = 0.958, *TLI* = 0.951, *SRMR* = 0.063. Like in the model with multiple mediators, career exploration self-efficacy did not impact on any LP dimension. In addition, compared to the model with multiple indicators, the associations between impact and identification were higher. This might be related to the removal of career decision-making self-efficacy from the model, endorsing its mediating effect on the relationship between impact and identification. Therefore, the alternative models corroborate the findings of the model with multiple mediators, meaning that the multicollinearity did not significantly affect the analyses (Figure 3).

The direct effect of impact on LP dimensions remained significant after the inclusion of the two career self-efficacy dimensions. Involvement remained strongly associated with impact. Nevertheless, the path from impact to identification lost magnitude, which is a consequence of the inclusion of the two career self-efficacy variables in the model. Indeed, an indirect effect of career decision-making self-efficacy was detected, though with a weak effect, std. *β* = 0.104[0.046; 0.173]. Like in the predictor-only model, distance was not associated with any LP dimensions. Therefore, the fourth hypothesis was partially corroborated.

## 4. Discussion and implications

This study examined the relationships between FTO, LP, and career self-efficacy in unemployed individuals. Four hypotheses were tested and were partially corroborated. According to the first hypothesis, unemployed individuals with higher rates of FTO are more likely to have more coherent LP. The findings showed that only one dimension of FTO (i.e., impact) was associated with LP’s identification and involvement; distance was not significantly related to any LP dimension. Thus, how one has current decisions and behavior impacted by the psychological future is a predictor of awareness about one’s intended future and engagement with activities and behavior toward one’s intended future. However, the coherence of one’s intended future, i.e., one’s LP, does not seem to be affected by how one perceives time distances into the future. This might suggest distance has mostly a cognitive nature, with no significant motivational implication, such as in the case of LP construction. Similar results were reported by Coscioni et al. (2023), who identified low correlations between distance and two other motivational variables, delay of gratification and career concern. The results seem to refute Nuttin and Lens (1985), who stated people who perceive distant events as upcoming events tend to be more committed and clearer about their future. However, both this study and the study of Coscioni et al. (2023) took place during the COVID-19 pandemic. This period was marked by a great extent of social isolation and uncertainty, which may have affected the way people perceive time distances. Future studies years after the pandemic is over still need to test whether distance does not show significant influence on motivational variables.

The second hypothesis stated unemployed individuals with higher rates of FTO are more likely to have more adaptive career self-efficacy. The findings showed that only one dimension of FTO (i.e., impact) was associated with career exploration and career decision making self-efficacy; distance was weakly associated with career exploration self-efficacy and did not correlate to career decision-making self-efficacy. Thus, how one has current decisions and behavior impacted by the psychological future is a predictor of individuals confidence in their abilities to explore and make decisions. However, the perceptions of decision-making ability does not seem to be affected by how one perceives time distances into the future. Thus, the results corroborate distance has little influence on motivational variables. On the other hand, the relationship of distance, although low, with exploration self-efficacy is a result that deserves attention. Exploration self-efficacy is a component that requires greater action from people (Taveira et al., 2023). Thus, in this case, the results corroborate Nuttin and Lens' (1985) theory. Nevertheless, given the low correlation, these implications are merely suggestions.

According to the third hypothesis, unemployed individuals with higher rates of career self-efficacy are more likely to have more coherent LP. The findings showed that only one dimension of career self-efficacy (i.e., career decision making) was associated with one of the LP dimensions (i.e., identification), though with a weak effect. Thus, how one perceives oneself as more or less capable to make decisions about their career is a predictor of awareness about one’s intended future. In a decision-making process, it is expected that people are able to identify and commit to a goal. Thus, greater self-confidence on decision-making expectedly influences more sharply how one identifies the intended future rather than how one acts accordingly. Quite possibly, no significant relationships of decision-making self-efficacy with involvement were identified due to the strong correlation between the two LP dimensions (i.e., identification and involvement). On the other hand, it can be speculated that exploration self-efficacy did not affect participants’ LP because unemployed people tend to be in disadvantaged social situations (e.g., Mühlhaus et al., 2021). This context may lead these people to prioritize any job opportunity rather than engaging in a process of exploration and deliberation (Lent et al., 2022).

The fourth hypothesis stated that career self-efficacy mediates the relationship between FTO and LP in unemployed people. The findings showed a mediating effect of only one career self-efficacy dimension (i.e., career decision-making self-efficacy) particularly on the relationship between one FTO dimension (i.e., impact) and one LP dimension (i.e., identification). The prevalence of non-significant relationships and reduced magnitudes might be related to the fact that the psychological future variables (i.e., FTO and LP) were measured using athematic scales (i.e., not associated with any particular life domain). In turn, self-efficacy was measured using a thematic scales focused on career. If the psychological future variables had also been measured using thematic scales focused on career, the mediation relationship might have been greater. Still, the results support the original idea of Marko and Savickas (1998), which indicates that the development of FTO is useful in promoting career planning.

This study provides valuable insights into the relationships between FTO, LP, and career self-efficacy in unemployed individuals. The findings underscore the importance of considering psychological future dispositions when developing career interventions for this group. Further research can delve deeper into these relationships, especially in the post-COVID-19 context, and explore practical approaches to integrating FTO dimensions into career intervention programs.

## 5. Practical implications

The degree to which people are influenced by their perceptions of their psychological future and the ease with which they can imagine the future are considered dispositions that vary from person to person depending on various factors (e.g., cultural, gender, etc.; Coscioni et al., 2020). Career interventions with unemployed people should consider that people’s dispositions regarding their psychological future influence how they construct their LP directly and indirectly, through the development of career self-efficacy. Thus, to favor the development of those dispositions regarding the psychological future, career interventions may include certain activities and approaches.

Supporting the recognition of personal resources and strengths (Ginevra et al., 2017) can help unemployed people to feel more able to pursue their future projects. In order to encourage people to think about the future, it may also be important to support them to set realistic short and medium-term goals. This could be done, for example, through the SMARTE (Specific, Measurable, Attainable, Realistic, Time-bound, Effect) goals activity. To carry out this activity, people should be invited to select one of their career goals, and define it in a S.M.A.R.T.E. way. That is, they should think, respectively, “What do you want to achieve? What is my goal?”; “How will I know that I have achieved my goal? What will have changed?”; “Will I have the necessary conditions to achieve the goal?”; “Why do I want to achieve this goal? Is it a reasonable and realistic goal to achieve? What barriers will I face? How?”; “How much time will I need to achieve my goal? What is my timeframe?”; “What value will arise from this? What effect will it have on me if I achieve this goal?.” Activities that allow monitoring of future career goals, may favor people’s life projects through their self-efficacy beliefs.

Career interventions with unemployed people could also invest in promoting self-efficacy in decision making (Atanásio et al., 2013). This does not mean that the exploration process is neglected, quite the opposite. As suggested by Taveira et al. (2023), one should firstly favor persistence and commitment to the exploration process. However, the self-efficacy beliefs resulting from this process should then be directed toward the action-taking process to help people develop further career decisions (e.g., Koen et al., 2010). For example, interventions that target the four sources of self-efficacy (i.e., performance accomplishments, vicarious learning, emotional arousal, verbal persuasion; Bandura, 1997) should be explored as there is evidence that they increase an individual’s career decision-making self-efficacy (Bullock-Yowell et al., 2012). In this exploration process, psychologists can encourage individuals to consider options that are congruent with their identity, but which had not previously been explored (Bullock-Yowell et al., 2012).

Since FTO is a disposition and not a state, these results prove the difficulty of supporting people in the condition of unemployment (Panari and Tonelli, 2022). Despite the complexity, these career interventions should include activities that support people to clearly define their future career and to feel more confident about their opportunities in the job search process. This tends to increase the likelihood of people finding a job congruent with their goals (Koen et al., 2010). Lastly, considering that the study took place during COVID-19 period, career interventions with the unemployed population may consider specific strategies to support the unemployed during crises like the pandemic, addressing self-efficacy issues and career planning in situations of uncertainty. In general, career intervention programs with unemployed people aimed at organizing their LP must necessarily include components of FTO. This means that all the activities and attitudes of the career counselors should favor the importance of planning the future.

## 6. Limitations and future studies

Although our findings are encouraging, there are some limitations that must be considered. First, the questionnaire was administered to the unemployed people at a public employment service. So, there is a chance that these results were biased by social desirability because the unemployed would want to show their best behavior in a context that they are using to help them find new work. Or on the other hand, they may be negatively biased, in the sense that respondents may in some way be dissatisfied with the support they receive from the entity. While partnerships with these entities are essential for research access to this population, it may be important to ensure a more neutral data collection context in the future. Secondly, the use of athematic measures to assess the psychological future of unemployed individuals might have affected the results and explain the low magnitude of the relationships of career self-efficacy to both FTO and LP. Future studies could study the relationship between career self-efficacy and thematic measures of the psychological future focused on career. Thirdly, the sample size was not powerful enough to detect significant parameters with an effect of *β* < 0.265. Bigger samples could have detected significant parameters with a weaker effect. Additionally, larger sample sizes would facilitate the implementation of a multigroup mediation model, enabling a better understanding of how sociodemographic variables (i.e., age and gender) mediate the relationship between psychological future variables and career self-efficacy. Fourth, it is important to acknowledge that the context of the COVID-19 pandemic may have influenced participants’ responses in this study. The uncertainty and social isolation during this period could have impacted perceptions of time distance and job searching. Therefore, the results should be interpreted in light of these exceptional conditions. Although the study had its limitations, it provided interesting insights into how unemployed people function in terms of self-efficacy and psychological future, as well as about the priorities for interventions with this population.

## 7. Conclusion

The results indicate that people who are highly impacted by the future tend to have more decision-making self-efficacy and, therefore, to have a more identified LP. In other words, there is a direct effect between FTO and LP, but also an indirect (i.e., mediating) effect between them through career self-efficacy beliefs. Globally, the study provides important insights regarding the psychological factors that can directly or indirectly impact careers prospects of unemployed individuals, as well as on the characteristics of career psychological interventions with this public. The relevance of including FTO components in career interventions aimed at supporting the organization of unemployed individuals’ LP stands out.

## Data availability statement

The raw data supporting the conclusions of this article will be made available by the authors, without undue reservation.

## Ethics statement

The studies involving humans were approved by the Committee for Research in Social Sciences and Humanities, Ethics Council of the University of Minho (CEICSH 002/2022). The studies were conducted in accordance with the local legislation and institutional requirements. The participants provided their written informed consent to participate in this study.

## Author contributions

AS, CC, and VC contributed to conception and design of the study. CC organized the database. VC performed the statistical analysis. CC and VC wrote the first draft of the manuscript and sections of the manuscript. AS and MT contributed to manuscript revision, read, and approved the submitted version. All authors contributed to the article and approved the submitted version.

## Footnotes

^1^The internal structure was assessed *via* confirmatory factor analysis with maximum likelihood robust estimator (MLR; Satorra and Bentler, 2001).
